# Occurrence and Multi-Locus Genotyping of *Giardia duodenalis* in Bamaxiang Pigs in Bama Yao Autonomous County of Guangxi Province, China

**DOI:** 10.3390/vetsci12121114

**Published:** 2025-11-22

**Authors:** Qiaoyu Li, Wenjing Zeng, Sifan Wang, Xuanru Mu, Hui Xu, Yange Lin, Mingxin Lv, Yilong Li, Xingang Yu, Yang Hong

**Affiliations:** 1 School of Animal Science and Technology, Foshan University, Foshan 528231, China; 13435773346@163.com (Q.L.); zengwenjing2020@163.com (W.Z.); 13535899565@163.com (S.W.); 15940815092@163.com (X.M.); xuhui990105@163.com (H.X.); 15016003403@163.com (Y.L.); lmx20020117@163.com (M.L.); muylyl20001110@163.com (Y.L.); 2 National Institute of Parasitic Diseases, Chinese Center for Diseases Control and Prevention (Chinese Center for Tropical Diseases Research), Key Laboratory of Parasite and Vector Biology, National Health Commission of the People’s Republic of China (NHC), World Health Organization (WHO) Collaborating Center for Tropical Diseases, National Center for International Research on Tropical Diseases, Shanghai 200025, China

**Keywords:** *Giardia duodenalis*, Bamaxiang pig, multilocus genotyping, zoonosis

## Abstract

Background: *Giardia duodenalis* is one of the most prevalent protozoan pathogens, commonly infecting a wide range of hosts including humans, livestock, companion animals, and wildlife globally. The Bamaxiang pig (*Sus scrofa domesticus*), a native livestock variety from China’s Guangxi region, holds significant importance in Bama Yao Autonomous County. This breed not only supports regional meat production systems but also fulfills dual roles as human companions and valuable subjects for biomedical investigations. The aim of present study was to investigate the prevalence of *G. duodenalis* and assess its genetic characteristics. A total of 311 fresh fecal samples were collected from three farms in Bama Yao Autonomous County. The presence and genetic diversity of *G. duodenalis* were determined by nested PCR and sequence analysis of the glutamate dehydrogenase (*gdh*), β-giardin (*bg*) and triose phosphate isomerase (*tpi*) genes. Results: The total occurrence rate of *G. duodenalis* in Bamaxiang Pigs was 17.36% (54/311). Among different age groups, suckling piglets exhibited the highest infection rate at 24.29% (17/70). The infection rates in the sows, fattening pigs, and weaned piglets were 20.88% (19/91), 14.10% (11/78), and 9.72% (7/72), respectively (*p* < 0.05). Phylogenetic analysis of sequences from three genetic loci identified two *G.duodenalis* genetic assemblages, namely assemblages A and E. Among them, assemblage A (*n* = 31) was the predominant genotype across the three farms, followed by the assemblage E (*n* = 21) and mixed assemblage A/E (*n* = 2) infections. Conclusions: This study represents the first report demonstrating that *G. duodenalis* infection is prevalent in Bamaxiang pigs, with variable positivity rates across different growth stages. The zoonotic assemblage A was the predominant assemblage, suggesting a potential risk of transmission to humans through close contact with this specific pig breed. The results provide basic data for controlling infections in Bamaxiang pigs.

## Simple Summary

*Giardia duodenalis* is a significant zoonotic intestinal parasite infecting a wide range of hosts worldwide. The Bamaxiang pig, an important indigenous breed in China, was investigated in this study for *G. duodenalis* occurrence and genetic characteristics. A total of 311 fecal samples from three farms in Bama Yao Autonomous County were analyzed by nested PCR, revealing an overall infection rate of 17.36% (54/311). Prevalence varied significantly (*p* < 0.05) among age groups: highest in suckling piglets (24.29%, 17/70), followed by sows (20.88%, 19/91), fattening pigs (14.10%, 11/78), and weaned piglets (9.72%, 7/72). Genetic analysis identified two assemblages: the zoonotic assemblage A predominated (*n* = 31), followed by assemblage E (*n* = 21), with two cases of mixed A/E infections. This first report of *G. duodenalis* in Bamaxiang pigs highlights a high prevalence of zoonotic assemblage A, suggesting potential public health risks and providing key data for control strategies.

## 1. Introduction

*Giardia duodenalis* (syn. *G. intestinalis*, *G. lamblia*) is one of the most prevalent protozoan pathogens affecting diverse mammalian hosts globally, with zoonotic transmission occurring between humans, domestic animals (such as goats and pigs), and wildlife species [1,2]. The parasite poses significant public health concerns and may lead to acute or chronic diarrhea, nausea, abdominal pain, vomiting, and weight loss [3]. Since the 1970s, giardiasis has been reported globally, and is recognized by the World Health Organization as a neglected disease posing a threat to human health [4]. It has been estimated that over 280 million individuals are affected by giardiasis globally each year [5].

The life cycle of *G. duodenalis* involves two distinct morphological forms: trophozoites that colonize the intestinal epithelium and cysts that persist in the environment. Susceptible animals are infected by the ingestion of contaminated water, food, or through contact with infected animals [3]. Based on genetic analysis, *G. duodenalis* can be divided into eight assemblages (A–H). Assemblages A and B have been reported in humans and other mammalian hosts, the remaining assemblages are host-specific: assemblages C and D are found mainly in canids, assemblage E in hoofed domestic and wild animals, assemblage F in cats, assemblage G in rodents, and assemblage H in pinnipeds [6].

Pigs are widely raised across the globe and are recognized as one of the most significant hosts for *G*. *duodenalis* infection [1,5]. The global prevalence of giardiasis in pigs is estimated at 9.1% (95% CI: 5.6–14.3), with a pooled molecular prevalence in China of 6.5% (95% CI: 6.0–7.0). To date, five distinct *G. duodenalis* assemblages (A–E) have been identified in pigs across China, with assemblage E being the most prevalent genotype [1]. However, a previous study conducted in the Guangxi Zhuang Autonomous Region reported assemblage A as the dominant genotype in local pig populations [7]. Notably, Guangxi features a subtropical climate and extensive karst plateau topography with well-developed underground river systems, where zoonotic *Giardia* cysts may contaminate groundwater and pose a potential public health risk.

As a native Chinese pig breed, the Bamaxiang holds distinction among indigenous purebred swine populations. The breed is known for its small size and distinctive two-sided black coat [8,9]. Compared to other pig breeds, it exhibits precocious puberty along with enhanced traits including meat quality, adaptability, and pathogen resilience. In addition to serving as both a commercially significant indigenous breed and a promising biomedical model, Bamaxiang pigs are also suitable as companion animals, demonstrating their remarkable versatility [10,11]. At present, there are no reports on the infection of *G. duodenalis* in Bamaxiang pigs. Therefore, this study investigated *G. duodenalis* infection in different age groups of Bamaxiang pigs across three large-scale farms in Bama Yao Autonomous County, Guangxi Province.

Due to the limitations of single-marker genotyping, certain *G. duodenalis* genotypes may remain unidentified. In recent years, multilocus genotyping (MLG) approaches involving the amplification of multiple conserved genes including beta-giardin (*bg*), triose phosphate isomerase (*tpi*), and glutamate dehydrogenase (*gdh*) have become the prevailing methodological standard for accurate assemblage discrimination. In the present study, we employed nested PCR targeting *bg*, *gdh*, and *tpi* for genotype identification. These findings provide fundamental data for the prevention and control of *G. duodenalis* in Bamaxiang pigs while also enhancing our understanding of the genetic diversity and zoonotic potential of this parasite.

## 2. Materials and Methods

### 2.1. Fecal Sample Collection

From May to July 2024, a total of 311 fresh fecal samples were systematically collected from three commercial Bamaxiang pig farms in Bama Yao Autonomous County. The Bamaxiang pigs in this study were housed in open-sided shed structures that provided partial exposure to the outdoor environment. Fecal samples were collected rectally from piglets and fattening pigs, while for sows, freshly voided feces were collected from the floor within 1–2 min. All collections were conducted by trained graduate researchers. A structured monitoring approach was implemented in sows, with each researcher assigned 5–10 pigs for continuous observation. Fecal specimens were specifically obtained from the aerobically exposed surface layer using sterile disposable gloves.

These samples comprised four distinct age cohorts: suckling piglets (*n* = 70; <21 days), weaned piglets (*n* = 72; 21–70 days), fattening pigs (*n* = 78; 71–180 days), and sows (*n* = 91; >180 days) [12]. Immediately following collection, each specimen was carefully placed into individual disposable plastic bags pre-labeled with essential information including the farm, pig age, and date of collection. All samples were promptly transported on ice to the laboratory and preserved in a 2.50% (*w*/*v*) potassium dichromate solution at 4 °C, with subsequent processing conducted within seven days post-collection.

### 2.2. Fecal DNA Extraction

Fecal samples were subjected to distilled water washes to remove potassium dichromate prior to DNA extraction [13]. DNA from each fecal sample was extracted from 200 mg portions using the E.Z.N.A.^®^ Stool DNA Kit (Omega Bio-tek, Norcross, GA, USA) as per standard protocols.

### 2.3. PCR Amplification

*G. duodenalis* was identified and genotyped by nested PCR amplification and sequencing of three loci (The *gdh*, *bg*, and *tpi*) (Table 1) [13]. PCR amplification was conducted in a 25 µL reaction mixture containing 12.5 µL of 2× Taq Master Mix (Dye Plus; Vazyme Biotech, Nanjing, China), 1 µL of forward and reverse primers (10 µM each), 2 µL of template DNA, and 8.5 µL of nuclease-free ddH_2_O. Amplified products were resolved by electrophoresis on a 1.20% agarose gel, visualized using a commercial nucleic acid stain (Biosharp Life Sciences, Beijing, China), then analyzed and documented utilizing the Azure™ c200 imaging platform (Azure™ c200, Dublin, CA, USA). Strict quality control measures were implemented, including concurrent runs of positive controls (reference *G. duodenalis* DNA) and negative controls (nuclease-free water) in every PCR batch to monitor contamination and amplification efficiency.

**Table 1:** PCR primers targeting *G. duodenalis* markers.

Gene	Primer Sequences (5′-3′)	Annealing Temperature (°C)	Predicted Fragment Size (bp)	Reference
*bg*	F1: AAGCCCGACGACCTCACCCGCAGTGC	55	515	[14]
	R1: GAGGCCGCCCTGGATCTTCGAGACGAC			
	F2: GAACGAACGAGATCGAGGTCCG			
	R2: CTCGACGAGCTTCGTGTT			
*tpi*	F1: AAATYATGCCTGCTCGTCG	57	530	[15]
	R1: CAAACCTTYTCCGCAAACC			
	F2: CCCTTCATCGGYGGTAACTT			
	R2: GTGGCCACCACYCCCGTGCC			
*gdh*	F1: TTCCGTRTYCAGTACAACTC	59	530	[16]
	R1: ACCTCGTTCTGRGTGGCGCA			
	F2: ATGACYGAGCTYCAGAGGCACGT			
	R2: GTGGCGCARGGCATGATGCA			

### 2.4. Sequencing and Phylogenetic Analysis

All positive PCR products were sent to Sangon Biotech (Shanghai, China) for commercial DNA sequencing. Sequence alignment was performed against the NCBI GenBank database using BLAST (National Center for Biotechnology Information, Bethesda, MD, USA). Analysis was conducted on https://blast.ncbi.nlm.nih.gov/Blast.cgi (accessed on 15 June 2025). The *bg*, *gdh*, and *tpi* loci sequenced from *Giardia* isolates (available in Supplementary Files S1–S3) were aligned against reference sequences retrieved from GenBank (see Supplementary Tables S2–S4). The genetic relationships of *G. duodenalis* were evaluated by building phylogenetic trees using maximum likelihood analysis in MEGA 7.0 software (version 7.0, accessed 25 June 2025). Tree stability was evaluated by performing 1000 bootstrap pseudoreplicates.

### 2.5. Statistical Analysis

The prevalence rates of *G. duodenalis* infections, along with their corresponding 95% confidence intervals (95% CIs), were determined. To examine variations in prevalence across different age groups, statistical analyses were performed using the Pearson’s chi-square test (χ^2^). This assessment was carried out in crosstabs within SPSS version 27.0 (IBM SPSS Inc., Chicago, IL, USA). A *p*-value < 0.05 was regarded as statistically significant for all analyses.

## 3. Results

### 3.1. Occurrence of G. duodenalis

Based on the PCR detection of any of the three conserved genetic markers (*bg*, *tpi*, and *gdh*), *G. duodenalis* was detected in 17.13% of samples (54/311; 95% CI: 13.10–21.60). *G. duodenalis* was detected in pigs from all age groups (suckling piglets, weaned piglets, fattening pigs and sows). The infection rate was highest in suckling piglets (24.29%, 17/70; 95% CI: 14.00–34.60), followed by sows (20.88%, 19/91; 95% CI: 12.40–29.40), fattening pigs (14.10%, 11/78; 95% CI: 6.20–22.00), and weaned piglets (9.72%, 7/72; 95% CI: 2.70–16.70) (*p* < 0.05) (Table 2).

### 3.2. Genotypes of G. duodenalis

Among the 54 *G. duodenalis*-positive samples, 29, 47, and 14 were positive for the *bg*, *gdh*, and *tpi* loci, respectively (Table S1). Notably, complete concordance across all three target genes (*bg*, *gdh*, and *tpi*) was observed in 7 samples (12.96%, 7/54), while 22 samples (40.74%, 22/54) showed positivity for two loci and 25 samples (46.30%, 25/54) were single-gene positive.

**Figure 1:**
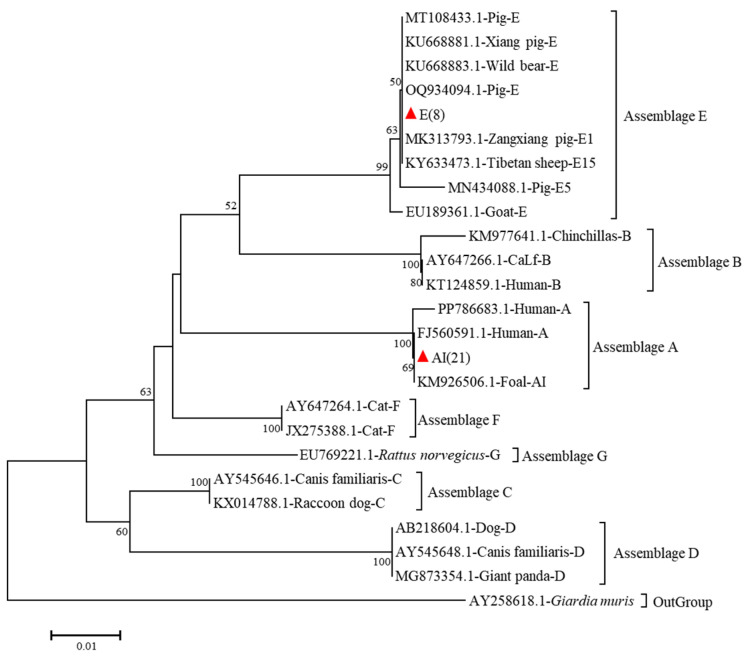
Phylogenetic relationships of *G. duodenalis* obtained from Bamaxiang pigs based on *bg* gene markers in Bama Yao Autonomous County, Guangxi Province, China. The phylogenetic reconstruction employed the Tamura–Nei nucleotide substitution model coupled with bootstrap resampling (1000 replicates). Distinct genotypes characterized in our investigation are indicated by filled crimson deltoid markers, while nodal support probabilities exceeding 50% bootstrap confidence are explicitly annotated.

Genetic analysis revealed the presence of two distinct *G. duodenalis* genotypes, specifically assemblages E and A, among the sampled specimens. At the *bg* locus, twenty-one isolates were genotyped as assemblage A (subtyped as AI), while eight isolates belonged to assemblage E. Assemblage A sequences (*n* = 21) exhibited >99% homology with human-derived sequence (GenBank accession number: PP786683.1), while assemblage E isolates (*n* = 8) matched 100% with pig-derived OQ934094.1 (Figure 1).

**Figure 2:**
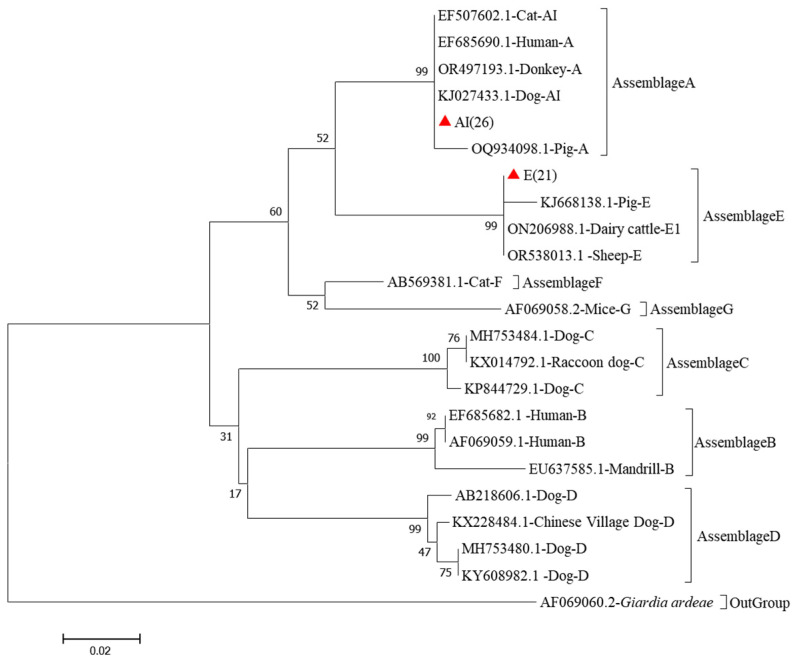
Phylogenetic relationships of *G. duodenalis* obtained from Bamaxiang pigs based on *gdh* gene markers in Bama Yao Autonomous County, Guangxi Province, China. The phylogenetic reconstruction employed the Tamura–Nei nucleotide substitution model coupled with bootstrap resampling (1000 replicates). Distinct genotypes characterized in our investigation are indicated by filled crimson deltoid markers, while nodal support probabilities exceeding 50% bootstrap confidence are explicitly annotated.

The *gdh* locus classified 26 isolates as assemblage A and 21 as assemblage E. Assemblage A sequences aligned closely (>99%) with KJ027433.1 from dogs in China, whereas assemblage E shared 99% homology with KJ668138.1 from pigs in China (Figure 2).

**Figure 3:**
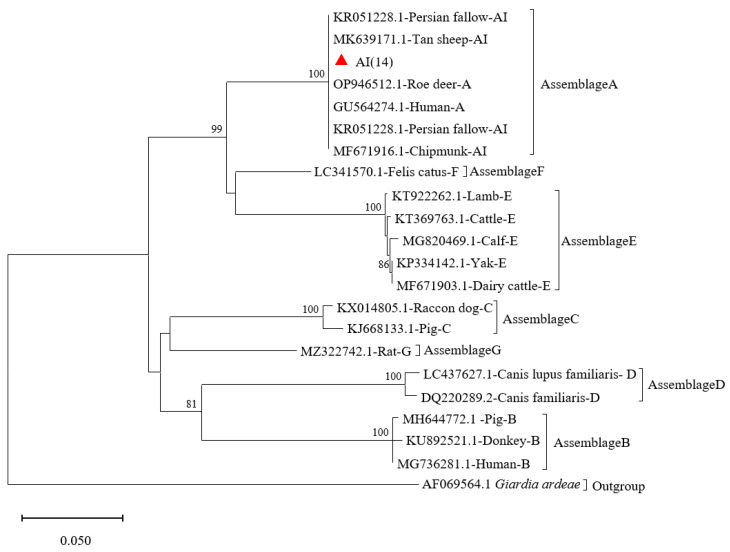
Phylogenetic relationships of *G. duodenalis* obtained from Bamaxiang pigs based on *tpi* gene markers in Bama Yao Autonomous County, Guangxi Province, China. Phylogenetic relationships of *G. duodenalis* obtained from Bamaxiang pig using *tpi* gene markers. The phylogenetic reconstruction employed the Tamura–Nei nucleotide substitution model coupled with bootstrap resampling (1000 replicates). Distinct genotypes characterized in our investigation are indicated by filled crimson deltoid markers, while nodal support probabilities exceeding 50% bootstrap confidence are explicitly annotated.

Sequencing analysis of the *tpi*-positive *G. duodenalis* isolates (*n* = 14) demonstrated consistent clustering within assemblage AI, exhibiting evolutionary conservation (>99% sequence identity) with the human-derived reference sequence GU564274.1 previously reported in China (Figure 3).

## 4. Discussion

*Giardia duodenalis* is a prevalent zoonotic gastrointestinal parasitic protozoan with significant veterinary and public health implications worldwide. The global frequency of giardiasis in pigs is estimated at 9.1% (95% CI: 5.6–14.3), with reported ranges from 0.6% to 66.4% worldwide [17]. With the exception of specific wild boar populations and diarrheic specimens, developing countries generally exhibit higher *Giardia* prevalence in domestic pig herds than developed countries or regions. The pooled continental prevalence estimates are: Africa (25.3%; 95% CI: 19.9–31.6), Asia (5.9%; 95% CI: 3.3–10.3), Europe (8.1%; 95% CI: 3.5–17.9), North America (23%; 95% CI: 0.7–92.4), and Oceania (31.1%; 95% CI: 26–36.7).

The Bamaxiang pig, a Chinese indigenous miniature breed, is economically valued for its unique meat nutrition while being utilized as an experimental model in research and increasingly kept as companion animals [10]. In this study, the overall infection rate of *G. duodenalis* in Bamaxiang pigs across three farms was 17.36% (54/311), which was lower than the domestic pig (*Sus domesticus*) in Jilin (45.0%, 27/60) [18], Fujian (26.9%, 195/725) [19], Shanghai (26.88%, 25/93) [20], and Guangdong (18.04%, 94/521) [21], but higher than that in Guigang (1.1%, 2/177), Nanning (10.8%, 23/213), Yulin (0.7%, 1/146) [7], Xiaogan (1.90%, 4/210), Wuhan (0.99%, 3/102), Xianning (0.68%, 1/148) [22], Tibet Autonomous Region (0.58%, 2/345) [23], Shaanxi 8% (45/560) [24], Yunnan 2.5% (5/200), Zhejiang 10.5% (13/124), Xinjiang 2.6% (21/801) [25], Taiwan 4.26% (6/141) [26], Shaanxi and Qinghai 6.2% (28/450) [27] in China. On a global scale, the infection rate in this study was higher than those reported in Denmark (14.0%, 120/856) [28] and South Korea (14.8%, 110/745) [29], but lower than that in Nigeria (25.4%, 53/209) [30]. The reported variation in *G. duodenalis* infection rates among pigs could be attributed to regional disparities [1], as well as variations in farm management practices [28], pig categories [1,27], sampling sizes [1], and seasonal factors [29]. The Bamaxiang pigs in this study were housed in open-sided shed structures that provided partial exposure to the outdoor environment. General hygiene at the farms was maintained at a satisfactory level, with workers regularly performing intensive cleaning of the pens using high-volume water rinsing. However, the overall management approach could be characterized as relatively extensive. Critically, these semi-open housing conditions allowed for occasional contact between the pigs and other animal species, including domestic dogs, cats, cockroaches, and other insects. The shared environment creates a potential risk of the mutual transmission of *Giardia* among these hosts [1,5]. To pinpoint the precise drivers behind the observed prevalence differences, more extensive epidemiological investigations with larger sample sizes are needed.

In the present study, the infection rate of *G. duodenalis* was 24.29% (17/70) in suckling piglets, 9.72% (7/72) in weaned piglets, 14.10% (19/91) in fattening pigs, and 20.88% (19/91) in sows. Suckling piglets and sows exhibited significantly higher infection rates than other age groups (*p* < 0.05). These findings align with previous research in Guangxi Province, which reported elevated prevalence in breeding pigs (5.1%, 9/175) and piglets (6.0%, 11/183) compared to fattening pigs (2.4%, 4/165) and conservation pigs (1.0%, 2/201) [7,27]. In contrast, another study in southern China observed a substantially higher infection rate (12.3%) in pigs aged 4–6 months than those aged 1–3 months (2.3%) [31]. Recent studies indicate that age-related prevalence variations may be attributed to multiple factors, including immune development, gut microbiota composition, nutritional status, and geographic isolation. Suckling piglets experience the highest infection intensity, likely due to their immature immune systems [28]. Porcine neonates demonstrate incomplete immunological maturation until approximately 28 days postnatally, resulting in diminished capacity to mount effective immune responses against infectious agents during this period [32]. Additionally, environmental stressors and nutritional deficiencies may further compromise their resistance to *G. duodenalis* infection [7].

To date, among the eight known *G. duodenalis* assemblages (A–H), six (A, B, C, D, E and F) have been detected in pigs worldwide, with assemblage E being predominant. In China, five assemblages (A, B, C, D, and E) have been reported in pig populations [1,17]. In the present study, two *G. duodenalis* strains, belonging to assemblages A and E, were detected in the sampled population. Among the 54 PCR-positive fecal samples from Bamaxiang pigs, target amplification yielded 47 (*gdh*), 29 (*bg*), and 14 (*tpi*) valid sequences, revealing notable differences in locus-specific amplification and typing efficiency. Further analysis showed complete concordance across all three genes (*gdh*, *bg*, and *tpi*) in 7 samples (12.96%), while 22 (40.74%) were positive for two loci and 25 (46.30%) exhibited single-gene positivity. This result is likely attributable to the varying substitution rates among *G. duodenalis* genetic loci, leading to differences in detection and typing resolution across targeted genomic regions [7,33]. As reported by Feng and Wielinga et al. [33,34], genetic loci in *Giardia* exhibit variation in substitution rates, leading to differences in resolution for parasite typing. For instance, the substitution rates for partial *SSU rRNA*, *bg*, *gdh*, and *tpi* genes were reported as 0.01, 0.03, 0.06, and 0.12 substitutions per nucleotide, respectively. In a related study, Shamsi et al. [35] evaluated the pooled prevalence of *G. duodenalis* in human samples and found that pooled prevalence and diagnostic accuracy were highest using the *tpi* gene (64.3%; 95% CI: 56.1–71.8%), followed by *gdh* (59.7%; 95% CI: 51.8–67.1%) and *bg* (58.3%; 95% CI: 49.8–66.3%). In some epidemiological studies, the highly conserved *SSU rRNA* gene served as the primary molecular marker for the initial detection and confirmation of *Giardia* in clinical samples, while many investigations employed multilocus genotyping (MLG) targeting *bg*, *gdh*, and *tpi* loci to obtain comprehensive genetic characterization [16,36,37]. In the present study, we implemented MLG-based analysis using *bg*, *gdh*, and *tpi* loci. However, substantial variations in amplification efficiency were observed across these targets—with *gdh* exhibiting the highest success rate, while *tpi* exhibited notably lower performance. This divergence between theoretical and practical detection efficacy may be influenced by several factors, including potential differences between the *Giardia* assemblages prevalent in animal reservoirs and those in our human cohort [37]. Furthermore, as reported by Shamsi et al. [37], these genes exhibit assemblage-specific preferences: the *gdh* gene showed superior sensitivity for assemblage B (59.5%), whereas *bg* had a slightly higher detection rate for assemblage A (41.6%). To enhance the robustness of our genotyping, we plan to refine our future approach by incorporating the *SSU rRNA* gene into the MLG scheme.

In the current study, assemblage A (*n* = 31, 57.40%) and assemblage E (*n* = 21, 38.89%) were identified in 54 positive samples. Assemblage A was the predominant genotype in the present study and was distributed across all age groups. The zoonotic assemblage A exhibits extensive host specificity, infecting many mammals, including humans, livestock and wildlife [38]. While assemblage E is predominantly associated with ruminant species, including sheep and cattle, epidemiological studies have documented a rising incidence of human infections [39,40]. In the prior molecular characterization of *G. duodenalis* isolates from Zangxiang pigs, both zoonotic assemblages B and E were detected, with assemblage E exhibiting as the predominant genotype across all sampled locations and age groups except adults [27]. Despite both Bamaxiang pigs and Zangxiang pigs being classified as Chinese indigenous miniature breeds, they exhibit distinct differences in the assemblage diversity and dominant genotypes of *G. duodenalis* infections. Additionally, our laboratory has previously investigated *G. duodenalis* infection in pigs [21], cattle [41], and goats [16] across different regions of Guangdong Province. Our findings indicate moderate prevalence rates of *G. duodenalis* infection among the studied animals, with goats exhibiting the highest infection rate (24.78%, 56/226), followed by cattle (18.85%, 69/366), and pigs (18.04%, 94/521). Interestingly, assemblage AI showed a higher prevalence than all other subtypes in pigs and cattle, mirroring results from Bamaxiang pigs, with no assemblage B detected in any of these livestock populations. The contrasting distribution of *G. duodenalis* assemblages between Bamaxiang pigs and Zangxiang pigs, alongside the consistent predominance of assemblage A in Guangdong livestock, suggests that host-specific factors may influence genotype prevalence, warranting further comparative studies.

Pigs serve as natural reservoir hosts for *G. duodenalis*, and their fecal specimens frequently contain substantial quantities of infectious cysts. These cysts persist in the environment, potentially contaminating water sources or facilitating indirect transmission, thereby posing a zoonotic risk to humans and animals [42]. Bama Yao Autonomous County in northwestern Guangxi Province is characterized by a subtropical climate and karst plateau topography [12]. Periodic flooding in the region further enhances microbial survival and spread. Notably, *G. duodenalis* (genotypes A and E) detected in local livestock may contaminate water systems via runoff from intensive farms. Traditional practices in this minority-inhabited region, such as consuming raw foods, homemade fermented beverages, and water-soaked rice noodles, could facilitate fecal–oral transmission. Improved farm waste management and community health education are needed to mitigate potential zoonotic disease risks.

## 5. Conclusions

This study revealed a 17.36% (54/311) prevalence of *G. duodenalis* in Bamaxiang pigs, with suckling piglets showing the highest infection rate (24.29%, *p* < 0.05), and characterized its genetic profile. Phylogenetic analysis identified two assemblages: the zoonotic assemblage A (predominant, *n* = 31) and assemblage E (*n* = 21), with two mixed A/E infection. To our knowledge, this is the first report of *G. duodenalis* genotyping in Bamaxiang pigs. The high prevalence of assemblage A suggests zoonotic transmission potential, highlighting implications for public health and pig management. However, limitations such as seasonal variability in infection rates were not examined. Future studies should expand sample collection across seasons and regions to fully elucidate the epidemiological patterns and associated risks.

## Figures and Tables

**Table 2:** Colonization frequency and genotypic distribution of *G. duodenalis* in Bamaxiang pigs of different age groups in Bama Yao Autonomous County, Guangxi Province, China.

Age (Days)	Sample Size (Number)	*G. duodenalis*		
		No. Positive	Assemblage (Number)	Prevalence %(95% CI)
Suckling piglets (<21 days)	70	17	AI (8) E (8) AI + E (1)	24.29% (14.00–34.60%)
Weaned piglets (21–70 days)	72	7	AI (4) E (3)	9.72% (2.70–16.70%)
Fattening pigs (71–180 days)	78	11	AI (8) E (3)	14.10% (6.20–22.00%)
Sows (>180 days)	91	19	AI (11) E (7) AI + E (1)	20.88% (12.40–29.40%)
Total	311	54	AI (31) E (21) AI + E (2)	17.13% (13.10–21.60%)

## Data Availability

The original contributions presented in this study are included in the article/Supplementary Materials. Further inquiries can be directed to the corresponding authors.

## Supplementary Materials

The following supporting information can be downloaded at: https://www.mdpi.com/article/10.3390/vetsci12121114/s1. Table S1. Multilocus characterization of *G. duodenalis* isolates based on *bg*, *gdh*, and *tpi* genes; Table S2. GenBank accession numbers of all *bg* gene reference sequences of *G. duodenalis* used for phylogenetic analysis; Table S3. GenBank accession numbers of all *gdh* gene reference sequences of *G. duodenalis* used for phylogenetic analysis; Table S4. GenBank accession numbers of all *tpi* gene reference sequences of *G. duodenalis* used for phylogenetic analysis; File S1. The raw sequences of the *bg* gene of *G. duodenalis* used for phylogenetic analysis; File S2. The raw sequences of the *gdh* gene of *G. duodenalis* used for phylogenetic analysis; File S3. The raw sequences of the *tpi* gene of *G. duodenalis* used for phylogenetic analysis.

## Abbreviations

*G. duodenalis*	*Giardia duodenalis*
Bp	Base pair
*SSU rRNA*	Small subunit rRNA
*bg*	β-Giardin
*gdh*	Glutamate dehydrogenase
*tpi*	Triose phosphate isomerase
PCR	Polymerase chain reaction
