# Supplementary material for: Occurrence and Multi-Locus Genotyping of Giardia duodenalis in Bamaxiang Pigs in Bama Yao Autonomous County of Guangxi Province, China
Source: Vet Sci. 2025 Nov 22;12(12):1114. doi: 10.3390/vetsci12121114 (PMC12737703; doi:10.3390/vetsci12121114)
Supplement: Supplementary file 1 [file vetsci-12-01114-s001.zip › Table S4. GenBank accession numbers of all tpi gene reference sequences of G. duodenalis used for phylogenetic analysis.pdf]

Table S4. GenBank accession numbers of all *tpi* gene sequences of *G. duodenalis* used for phylogenetic analysis (Figure 4), and associated information.

GenBank ID	Genotype	Origin	Country	Assemblage
KR051228.1	AI	Persian fallow	China	Assemblage A
MK639171.1	AI	Tan sheep	China	Assemblage A
OP946512.1	A	Roe deer	Italy	Assemblage A
GU564274.1	A	Human	China	Assemblage A
KR051228.1	AI	Persian fallow	China	Assemblage A
MF671916.1	AI	Chipmunk	China	Assemblage A
LC341570.1	F	Felis catus	Japan	Assemblage F
KT922262.1	E	Lamb	China	Assemblage E
KT369763.1	E	Cattle	China	Assemblage E
MG820469.1	E	Calf	USA	Assemblage E
KP334142.1	E	Yak	China	Assemblage E
MF671903.1	E	Dairy cattle	China	Assemblage E
KX014805.1	C	Raccon dog	China	Assemblage C
KJ668133.1	C	Pig	China	Assemblage C
MZ322742.1	G	Rat	Austria	Assemblage G
LC437627.1	D	Canis lupus familiaris	Japan	Assemblage D
DQ220289.1	D	Canis familiaris	Australia	Assemblage D
MH644772.1	B	Pig	Nigeria	Assemblage B
KU892521.1	B	Donkey	China	Assemblage B
MG736281.1	B	Human	China	Assemblage B
AF069564.1	not available	Blue heron	Australia	Outgroup
