# Supplementary material for: Occurrence and Multi-Locus Genotyping of Giardia duodenalis in Bamaxiang Pigs in Bama Yao Autonomous County of Guangxi Province, China
Source: Vet Sci. 2025 Nov 22;12(12):1114. doi: 10.3390/vetsci12121114 (PMC12737703; doi:10.3390/vetsci12121114)
Supplement: Supplementary file 1 [file vetsci-12-01114-s001.zip › Table S2. GenBank accession numbers of all bg gene reference sequences of G. duodenalis used for phylogenetic analysis.pdf]

Table S3. GenBank accession numbers of all *bg* gene sequences of *G. duodenalis* used for phylogenetic analysis (Figure 3), and associated information.

GenBank ID	Genotype	Origin	Country	Assemblage
OQ934094.1	E	Pig	China	Assemblage E
KU668881.1	E	Xiang pig	China	Assemblage E
MT108433.1	E	Pig	Spain	Assemblage E
KU668883.1	E	Wild bear	China	Assemblage E
MK313793.1	E1	Zangxiang pig	China	Assemblage E
EU189361.1	E	Goat	Spain	Assemblage E
MN434088.1	E5	Pig	China	Assemblage E
KY633473.1	E15	Tibetan sheep	China	Assemblage E
KM977641.1	B	Chinchillas	China	Assemblage B
AY647266.1	B	Calf	Italy	Assemblage B
KT124859.1	B	Human	Malaysia	Assemblage B
AY647264.1	F	Cat	Italy	Assemblage F
JX275388.1	F	Cat	China	Assemblage F
FJ560591.1	A	Human	France	Assemblage A
PP786683.1	A	Human	China	Assemblage A
KM926506.1	AI	Foal	Belgium	Assemblage A
EU769221.1	G	*Rattusnorvegicus*	Sweden	Assemblage G
AY545646.1	C	Canis familiaris	Italy	Assemblage C
KX014788.1	C	Raccoon dog	China	Assemblage C
AB218604.1	D	Dog	Japan	Assemblage D
AY545648.1	D	Canis familiaris	Italy	Assemblage D
MG873354.1	D	Giant panda	China	Assemblage D
AY258618.1	not available	Laboratory mouse	Canada	OutGroup
