# Supplementary material for: Occurrence and Multi-Locus Genotyping of Giardia duodenalis in Bamaxiang Pigs in Bama Yao Autonomous County of Guangxi Province, China
Source: Vet Sci. 2025 Nov 22;12(12):1114. doi: 10.3390/vetsci12121114 (PMC12737703; doi:10.3390/vetsci12121114)
Supplement: Supplementary file 1 [file vetsci-12-01114-s001.zip › Table S3. GenBank accession numbers of all gdh gene reference sequences of G. duodenalis used for phylogenetic analysis.pdf]

Table S2 GenBank accession numbers of all *gdh* gene sequences of *G. duodenalis* used for phylogenetic analysis (Figure 2), and associated information.

GenBank ID	Genotype	Origin	Country	Assemblage
EF507602.1	AI	Cat	Brazil	Assemblage A
EF685690.1	A	Human	USA	Assemblage A
OR497193.1	A	Donkey	China	Assemblage A
KJ027433.1	AI	Dog	China	Assemblage A
OQ934098.1	A	Pig	China	Assemblage A
KJ668138.1	E	Pig	China	Assemblage E
ON206988.1	E1	Dairy cattle	China	Assemblage E
OR538013.1	E	Sheep	China	Assemblage E
AB569381.1	F	Cat	Japan	Assemblage F
AF069058.1	G	Mice	Australia	Assemblage G
MH753484.1	C	Dog	South Korea	Assemblage C
KX014792.1	C	Raccoon dog	China	Assemblage C
KP844729.1	C	Dog	Australia	Assemblage C
EF685682.1	B	Human	USA	Assemblage B
AF069059.1	B	Human	Australia	Assemblage B
EU637585.1	B	Mandrill	Italy	Assemblage B
AB218606.1	D	Dog	Japan	Assemblage D
KX228484.1	D	Chinese Village Dog	Romania	Assemblage D
MH753480.1	D	Dog	South Korea	Assemblage D
KY608982.1	D	Dog	Germany	Assemblage D
AF069060.2	not available	Blue heron	Australia	Outgroup
